# The influence of stereotype threat on immigrants: review and meta-analysis

**DOI:** 10.3389/fpsyg.2015.00900

**Published:** 2015-07-08

**Authors:** Markus Appel, Silvana Weber, Nicole Kronberger

**Affiliations:** ^1^Department of Psychology, University of Koblenz-LandauLandau, Germany; ^2^Department of Education and Psychology, Johannes Kepler University of LinzLinz, Austria

**Keywords:** stereotype threat, immigrants, meta-analysis, identity

## Abstract

In many regions around the world students with certain immigrant backgrounds underachieve in educational settings. This paper provides a review and meta-analysis on one potential source of the immigrant achievement gap: stereotype threat, a situational predicament that may prevent students to perform up to their full abilities. A meta-analysis of 19 experiments suggests an overall mean effect size of 0.63 (random effects model) in support of stereotype threat theory. The results are complemented by moderator analyses with regard to circulation (published or unpublished research), cultural context (US versus Europe), age of immigrants, type of stereotype threat manipulation, dependent measures, and means for identification of immigrant status; evidence on the role of ethnic identity strength is reviewed. Theoretical and practical implications of the findings are discussed.

## Introduction

In most nations, students with an immigration background score lower in achievement tests than non-immigrant students, and they leave school earlier (OECD, 2010). This status quo is particularly troublesome as the percentage of children and adolescents with an immigrant background is growing in many countries around the world, and immigration will likely be an even more prevalent phenomenon in the future (OECD, 2013). Consequently, the immigrant achievement gap is on the agenda of politicians, the general public, and social scientists alike.

Many immigrant groups are faced with negative achievement stereotypes in the countries they live in; these negative achievement stereotypes typically address those immigrant groups that indeed underperform. Our focus lies on the consequences of being exposed to a negative group stereotype regarding cognitive performance. According to stereotype and social identity threat theory and research, salient negative stereotypes can undermine the performance of negatively stereotyped group members due to an extra pressure not to fail (Steele and Aronson, 1995; Steele et al., 2002; Inzlicht and Schmader, 2012). Most of the experimental evidence on stereotype threat is based on women (stereotype: low ability in numerical domains) and African Americans (stereotype: low intellectual ability). Does stereotype threat affect the performance of immigrant students? In order to clarify the influence of stereotype threat on immigrants, the present article provides the first comprehensive review and meta-analytic summary of research (published and unpublished) on stereotype threat effects among this population.

### Immigration, Achievement, and Stereotype Threat

Stereotype threat is conceived as a state of psychological discomfort that is thought to arise when individuals are confronted with a negative stereotype about their own group in a situation in which the negative stereotype could be confirmed (Steele and Aronson, 1995; Steele et al., 2002). According to an integrative model of stereotype threat (Schmader et al., 2008; Schmader and Beilock, 2012) this state is characterized by the interplay of a physiological stress response, increased monitoring of the performance situation, and the regulation of negative thoughts and emotions. These processes consume working memory capacity, which is unavailable for the task at hand. The reduced working memory in turn leads to underperformance in cognitively challenging tasks (e.g., Schmader and Johns, 2003; Beilock et al., 2007). Stereotype threat is best known for its influence in testing situations. In line with the theoretical framework (e.g., Steele, 1997; Steele et al., 2002) several recent empirical studies furthermore connected stereotype threat to poorer learning and disidentification from school (e.g., Rydell et al., 2010; Appel et al., 2011; Taylor and Walton, 2011; Appel and Kronberger, 2012).

Stereotype threat theory and research has put little emphasis on the distinction of stereotyped groups. Indeed, many of the main findings have been demonstrated for samples of women as well as for samples of African Americans, despite important differences in stereotype content and breadth (Shapiro and Neuberg, 2007; Logel et al., 2012). Although Latinos in the US and immigrant groups in other countries outside the US are regularly mentioned in theoretical pieces and overview papers as potential targets of stereotype threat (cf. Inzlicht and Schmader, 2012), empirical studies are rare and an integrative review is missing. The passing attention paid to this group is particularly noteworthy in view of the overwhelming number of stereotype threat studies published in recent years. In stark contrast to this neglect are the systematic detrimental effects suffered by immigrants in educational systems around the globe. In most OECD countries, first generation immigrants (i.e., foreign-born immigrants) achieve lower scores in ability tests than students without immigration backgrounds. Second generation immigrants (i.e., immigrated before the age of six or at least one parent foreign-born) tend to score higher than first generation immigrants, but still fall short of non-immigrants (OECD, 2010). While language problems and low socio-economic status may be responsible for large parts of the achievement gap, a substantial part of the variance remains to be explained. Thus, it is of high interest to examine whether stereotype threat affects the cognitive performance of diverse immigrant groups in the same way as other minorities. If stereotype threat theory and research applied to immigrants, these stereotypes could prevent immigrants to perform up to their abilities (Steele and Aronson, 1995; Steele et al., 2002), they could inhibit them at times of preparation and learning (Rydell et al., 2010; Appel and Kronberger, 2012), and finally could contribute to a disidentification from school and academic achievements (Steele, 1992, 1997). Before presenting an overview on the current meta-analysis, in the following we discuss some of the difficulties, challenges, and open questions for immigration research and the implications these entail for our meta-analysis.

### The (Special?) Case of Immigrants

An important difficulty for stereotype threat research when applied to the field of immigrants is that many, but not all immigrant groups are faced with negative achievement stereotypes. In the past five decades, the majority of immigrants to the US have originated from either Asia or Latin America (US Department of Homeland Security, 2013). However, while Hispanic Americans are likely to be met with negative stereotypes in academic contexts (e.g., Nadler and Clark, 2011), Asian immigrants often are met with even superior expectations (e.g., Shih et al., 1999; Cheryan and Bodenhausen, 2000; Shih et al., 2002). Similarly, Northern and Western Europe – once a region where numerous people migrated from (cf. Hatton and Williamson, 1994) – over the past half a century has become an important destination for immigration, attracting people from Southern Europe, Turkey, Northern Africa, and former overseas colonies (e.g., Institute National de la Statistique et des Etudes Economiques [INSEE], 2013). Again, the stereotypic expectations directed at the various groups differ. While Turks in Germany, for example, are seen as “not willing to adapt” and “underdeveloped,” Italians are regarded as “well educated” (Jäckle, 2008); in Spain, immigrants from Latin America are stereotyped as being “lazy,” while Chinese immigrants have the image of being “hard working” and “smart” (Enesco et al., 2005). The examples not only illustrate the differential valence of stereotypes directed at different groups of immigrants, but also highlight that the content of stereotypes varies (Lee and Fiske, 2006). While some stereotypes concern cognitive and intellectual ability, others address aspects such as the willingness to integrate or diligence.

Due to the heterogeneity in stereotype valence and content, it is difficult to draw a coherent picture of the influence of stereotype threat on immigrants in general. However, it seems safe to say that Latin Americans in the US and Spain (Enesco et al., 2005; Tenenbaum and Ruck, 2007), and immigrants from Turkey, the Maghreb region, and the Balkan in Northern and Western Europe are likely to be characterized as underachieving at school (Verkuyten and Kinket, 1999; Kahraman and Knoblich, 2000; Jäckle, 2008). Accordingly, standardized testing suggests that it is exactly these groups that are most likely to show impaired performance compared to non-immigrants or more positively stereotyped immigrant groups (OECD, 2010). These are the groups this meta-analysis focuses on. African Americans are not included in the current meta-analysis because their history in the US dates further back in time and is different from other more recent immigrant groups, due to 300 years of slavery and another 100 years of post-slavery exploitation. This group also has been addressed already in great detail by prior stereotype threat theory and research (for an empirical overview, see Nguyen and Ryan, 2008).

A further challenge for research is that immigration status is a more complex and fuzzy category than other more visible and stable stereotype-relevant characteristics, such as gender or skin color. While it is extremely difficult for a man to ever count as a woman (or to be considered male and female at the same time), it is possible for many groups of immigrants to merge into the respective recipient culture (while potentially remaining identified with the culture of origin). This acculturation process may be easier for some groups of immigrants than for others, whereby the differential nature of stereotypes directed at them might be one reason for their difficulties. However, it is possible that due to the acculturation process at some point they are regarded, and regard themselves, as first and foremost belonging to the residence country and not any more as an immigrant^1^. This categorical malleability entails the question of how to define immigration status in empirical research; it has been suggested that being an immigrant is not only an objective characterization, but also, and even more importantly so, a subjective state of ethnic identification (Deaux, 1996).

Furthermore, and in an attempt to acknowledge the complex nature of defining immigrants, research has described the psychological consequences of migrating from one culture to the other, or being born as a member of an immigrant group along the lines of two identity dimensions (e.g., Berry, 1997; Phinney et al., 2001). One dimension is the attachment to one’s ethnic background of provenance, which includes the exploration of cultural practices of the culture of origin and the commitment to this cultural group. The second dimension is the attachment to one’s culture of residence. Both dimensions are considered to be conceptually independent (Berry, 1990, 1997; Liebkind, 2001; Phinney et al., 2001). Subject to the identity strength on both dimensions, four different “acculturation profiles” have been described: *assimilation* (low ethnic origin identity, high residence culture identity), *separation* (high ethnic origin identity, low residence culture identity), *marginalization* (low ethnic origin identity, low residence culture identity), and *integration* (high ethnic origin identity, high residence culture identity). *Bicultural identity integration* (Benet-Martinez and Haritatos, 2005; Nguyen and Benet-Martínez, 2010) is an alternative conceptualization, in which different ways to deal with the two potential group identities (ethnic origin and residential background) are distinguished: for some individuals, both identities may be perceived as compatible and overlapping, for others, both identities can be perceived as non-overlapping and a potential source of conflict.

Group identification is important because of its psychological consequences. Immigration research indicates, for example, that ethnic identification can buffer the negative effects elicited by societal devaluation and rejection of immigrants (e.g., Romero and Roberts, 2003; Umaña-Taylor and Updegraff, 2007; Armenta and Hunt, 2009; Umaña-Taylor et al., 2012). Similarly, ethnic identification is likely to play an important role when it comes to stereotype threat. Stereotype threat has been conceived as a result of a cognitive imbalance between the concept of self, the concept of a group, and the concept of an ability domain (Schmader et al., 2008). Stereotype threat occurs when individuals identify with a group (positive association between self and group) and identify with an ability domain (positive association between self and ability domain), while a negative stereotype suggests a negative connection between one’s group and the domain at hand. Prior research on stereotype threat has frequently addressed the moderating role of domain identification, that is, the self-domain link (for a review see Nguyen and Ryan, 2008). The negative effects of stereotype threat are more pronounced when individuals identify positively with the domain in question, either because it is part of their chronic self-concept (e.g., Aronson et al., 1999) or due to a situational prime of ego-involvement (Spencer et al., 1999)^2^. The self-group link has received much less attention, although the stereotype threat process model (Schmader et al., 2008; Schmader and Beilock, 2012) posits that stereotype threat effects are particularly strong when the connection to one’s group is highlighted by a situational prime; stereotype threat effects are also supposed to grow with the extent to which individuals identify with their stereotyped group. The empirical results on the self-group link are somewhat mixed. The threat increasing function of group identification has been demonstrated for women (Schmader, 2002) and African Americans (Ho and Sidanius, 2010), but other findings suggest that a strong identity might buffer stereotype threat effects among both groups (McFarland et al., 2003; Eriksson and Lindholm, 2007).

Extending the scope of stereotype threat theory to immigrants leads to the necessity of exploring the (potentially special) conditions and underlying mechanisms that apply for this particular target group. Overall it seems that definitions of group membership and processes of group identification may be more complex and less clear cut for immigrants than for other groups addressed by stereotype threat research. As a consequence, open questions abound on how and when ethnic identification affects intellectual performance. Moreover, there is a need to inspect the role of identification with the culture of residence (or a compatible and overlapping bicultural identity, Benet-Martinez and Haritatos, 2005).

### Rationale and Overview

Stereotype threat is a widely studied psychological phenomenon with potentially large implications for educational practice and related policies (cf. Cohen et al., 2012). Immigrants are frequently claimed to be one of the target groups of stereotype threat, but a systematic overview of available studies is missing. Despite the existence of a number of meta-analyses on stereotype threat, as yet, no meta-analysis has explicitly addressed the influence of stereotype threat on immigrants. Two meta-analyses were presented by Walton and Cohen (2003), providing results on the stereotype lift phenomenon, and on the relationship between ability and performance among various stereotyped and non-stereotyped groups (Walton and Spencer, 2009). Their emphasis, however, had not been on a comparison between differences among individuals under varied stereotype threat conditions, and no meta-analytic results were provided for immigrants. Nguyen and Ryan (2008) focused on stereotype threat effects, but no differentiation between African American and Latino American participants was made. Finally, Nadler and Clark (2011) examined stereotype threat effects among African Americans and Latinos in the US, but their sample relating to the latter group involved only four applicable studies (see Results) and stereotype threat research with immigrants in countries outside the US was not examined.

Beyond a quantification of the average effect size of stereotype threat research dealing with immigrants, we were interested in potential moderation effects. Heterogeneity in effect size often not only results from theoretically important variation, but also from characteristics of the samples included and from research practices (and potential related biases). To address the latter aspect, our first moderator was the publication status of the study. In a recent overview on stereotype threat research dealing with the math performance of female children and adolescents (Ganley et al., 2013), a null effect for unpublished research was reported. Thus, an analysis of moderation effects of publication status could provide a hint at potential publication bias.

We further examined whether results differed between studies with Latinos in the US versus different immigrant groups in European countries. Although stereotype threat generally got demonstrated in many countries, so far the large majority of studies has been conducted in the US. Cultural differences and variations in stereotype content might yield important differences in the threat experience and the effects on performance (cf. Eriksson and Lindholm, 2007). Thus, it was one of our main goals to identify whether or not stereotype threat effects on immigrants differed between cultures.

Research on stereotype threat is often based on undergraduate volunteers. We analyzed whether the study samples consisted of undergraduates or other samples like children and adolescents, because the experience of undergraduates might not be readily transferable to other samples (cf. Ganley et al., 2013).

We additionally analyzed whether different stereotype threat-activating cues, as defined by Nguyen and Ryan (2008), were used. In line with these authors, the following three categories were distinguished: *blatant* (explicit statement about the inferiority of one group, e.g., “women score lower in math than men”), *moderately explicit* (statement about subgroup differences in performance, but the direction of the differences is left open, e.g., “this test has shown gender differences in the past”), and *indirect and subtle* (i.e., no statement about subgroup differences; instead, the context of tests, test takers’ subgroup membership, or test taking experience is manipulated, e.g., test is “diagnostic” versus “not diagnostic”).

We were further interested in the tasks to assess performance. Are the results obtained equivalent for non-verbal tasks that assess basic cognitive abilities on the one hand (such as Raven’s progressive matrices) or tests that include more knowledge-based tasks on the other hand (such as GRE-typed measures)?

Finally, we closely inspected available studies for their way to assess immigrant group status. One method consists of participants self-identifying themselves as members of a certain immigrant group. However, another frequent option is to identify immigrants via demographic characteristics like the place of birth of themselves, their parents, or their grandparents. By employing the latter method, rather than self-categorization, individuals could be ascribed an immigrant background status, although they would self-define to belong to the mainstream culture. These individuals might be unaffected by a stereotype threat manipulation that targets their ethnic group of origin, due to the weak self-group link. As a consequence, studies that used methods other than self-identification might yield weaker effects than studies that relied on self-identification. Beyond immigrant status categorization, we were particularly interested in identity aspects, as previous research suggests competing predictions on the role of ethnic identity strength.

## Materials and Methods

### Literature Search and Selection Criteria

To identify relevant studies a literature search was conducted in December 2012, which was repeated in December 2013 and in March 2015. We searched the databases *PsycInfo, ERIC, SocIndex, Psyndex*, and *Psychology and Behavioral Sciences Collection* for studies that contained the terms “stereotype threat” or “social identity threat” and at least one of the terms migra^∗^ immigr^∗^, Latin^∗^, Hispanic^∗^, or Turk^∗^ (asterisk as a placeholder for different word endings)^3^. Our database search for literature published until 2014 resulted in 120 references. In addition, *google scholar* was searched for documents that contained a combination of the terms “stereotype threat,” “immigrant,” or “migrant,” and “experiment.” We further inspected the reference lists of book chapters and review articles for further studies. Finally, we asked for additional published or unpublished studies through e-mailing lists.

Full texts for potentially relevant studies were retrieved. We included studies that met the following criteria (inclusion/exclusion criteria): first, participants or a distinct subgroup of participants were members of a group with a recent and ongoing immigration history, such as Latinos in the US or Arab immigrants in Europe (because the history of African Americans is much older and intertwined with the complexities of race and skin color, this group was not included). Thus, studies that manipulated the salience of a stereotype about immigrants, but focused exclusively on non-immigrants did not meet this criterion (e.g., Chatard et al., 2008). Likewise, studies that did not report separate results for immigrants and other groups, most notably studies that reported combined average scores for Latino and African American participants were not included (e.g., Howard and Anderson, 2010). Second, the activated immigrant stereotype needed to be negative. This excluded research on the consequences of stereotypes regarding Asian immigrants (e.g., Cheung and Schmader, unpublished data), as widely held stereotypes regarding the cognitive performance of Asians are rather positive (e.g., Cheryan and Bodenhausen, 2000; Shih et al., 2002). Third, the study had to be experimental and had to follow the stereotype threat or social identity threat family of experimental treatments. Immigrant participants were supposed to be randomly assigned to two or more experimental groups. The conditions differed with respect to the salience of a negative stereotype addressing the immigrant group, the supposed relevance of the negative stereotype for an upcoming task (relevance), or the presence/absence of stimuli that signal non-belonging or belonging to a domain or society at large. Fourth, a measure of cognitive performance served as a dependent variable. Fifth, we inspected all studies for the quality of the applied methods and measures. This included an analysis of the operationalization of the independent variable and the dependent measures. Specifically, theory-based preconditions for stereotype threat to occur were inspected, such as sufficient domain identification and substantial task difficulty (Schmader et al., 2008).

We identified 18 texts (published and unpublished articles and dissertations) containing 21 experiments that met our criteria. In addition to 19 English-language texts, one report was written in German, and one in French (both languages were intelligible to us). In two cases the identical study was used for two separate journal articles. These results were included in our analysis only once. Thus, our final sample consisted of 19 experiments, 10 of which were unpublished (**Table 1**).

**Table 1:** Characteristics of the studies included in the meta-analysis.

Study no.	Study	P	Immigrant group	Manipulation of stereotype or social identity threat	Dependent measure	DV group
1	Appel (2012)	Yes	Immigrants of different origin in Austria	Anti-immigrant or neutral political party posters were shown in-between two intelligence tests	CFT	0
2	Armenta (2010)	Yes	Latino/as in the US	Test characterized either as diagnostic of intelligence and sensitive to ethnic differences or test purpose stated to be computerized versus paper-pencil	Math test (algebra, geometry, similar to GRE)	1
3	Berjot et al. (2011)	Yes	North Africans in France	Test characterized either as diagnostic of intelligence or as a memory test. Ethnicity asked before or after test.	Memory test (Rey-figure)	0
4	Chateignier et al. (2009)	Yes	Arabs in France	Test characterized either as diagnostic of intelligence or as characterized as a memory test in development	Verbal ability items (GRE)	1
5	Flanagan (2009)	No	Latino/as in the US	Classroom notes were shown in order to remind participants of ethnic inequalities or neutral condition	Verbal ability items (similar to GRE)	1
6	Froehlich et al. (in revision)	No	Turks in Germany	Test characterized either as diagnostic of verbal intelligence or as test under development	Verbal ability items (similar to GRE)	1
7	Gonzales et al. (2002)	Yes	Latino/as in the US	Test characterized either as diagnostic or as non-diagnostic of mathematical and spatial abilities	Mathematical and spatial ability (Wonderlic test)	1
8	Hollis-Sawyer and Sawyer (2008)	Yes	Latino/as in the US	Test characterized either as diagnostic of ‘general ability’ or as an interest measure	CFT	0
9	Klein et al. (2007)	Yes	Sub-Saharan Africans in Belgium	Test characterized as an entrance test for prestigious jobs. It was or was not further stated that Africans were found to underperform in this test	CFT	0
10	Mok et al. (in preparation)	No	Turks in Germany	Introduction stated that test has (not) revealed performance differences between Germans and Turks in the past	Verbal ability items (GRE)	1
11	Pellegrini (2005)	No	Latinas in the US	Test characterized either as diagnostic or as non-diagnostic of “true abilities”	WAIS (subtests letter-no., similarity, block, arithmetic)	1
12	Salinas (1998), Study 1	No	Latino/as in the US	Participants were or were not asked to rate the “level of bias” of the test prior to the crucial test part. Moreover, participants were or were not applied to electrodes and connected to a pseudo “effort meter”	Verbal ability items (GRE, computerized)	1
13	Salinas (1998), Study 2	No	Latino/as in the US	Participants were or were not asked to rate the “level of bias” of the test, prior to the crucial test part	Verbal ability items (GRE)	1
14	Schmader and Johns (2003), Study 2	Yes	Latino/as in the US	Task characterized either as predictive of intelligence and meant to establish group-wise norms or no such information	Working memory test	0
15	Schultz et al. (unpublished manuscript), Study 1	No	Latino/as in the US	Introduction mentioned previous underperformance of Latinos and participants had to list three reasons for that. In the control condition neither was this information given nor the task required	Verbal ability items (similar to GRE)	1
16	Schultz et al. (unpublished manuscript) Study 2	No	Latino/as in the US	Same as in Schultz et al. (unpublished manuscript), Study 1	Verbal ability items (similar to GRE)	1
17	Schultz et al. (unpublished manuscript) Study 3	No	Latino/as in the US	Same as in Schultz et al. (unpublished manuscript), Study 1	Verbal ability items (similar to GRE)	1
18	Stünzendörfer (2006)	No	Turks in Germany	Test characterized either as an ability test that shows differences between immigrants and non-immigrants or as a test non-diagnostic of abilities	Standard Progressive Matrices (Raven, 1958)	0
19	Wicherts et al. (2005)	Yes	Immigrants of different origin in the Netherlands	Test characterized either as an intelligence test and ethnicity items placed before the DV or test not characterized as an intelligence test and ethnicity items placed after DV	Dutch intelligence test (adapted), numerical, abstract reasoning, verbal reasoning	1


*P, Published?; CFT, “Culture Fair Test” (based on Cattell, 1940); WAIS, Wechsler Adult Intelligence Scale; DV, Dependent variable: 0, non-verbal fluid intelligence or memory tasks, 1, GRE-like measures (verbal, maths).*

### Effect Size Coding and Procedure

All three authors read and coded all available studies. A coding sheet was developed to gather the relevant information. Discrepancies between the judgments were very rare and resolved through discussions. Due to the fact that the examined datasets involved a comparison between two groups – stereotype threat high or low – the standardized mean difference was chosen as the effect size measure (Cohen’s *d*). When available, the effect size calculations were based on descriptive data (*M*s, *SD*s, *n*s), when unavailable, formulas to calculate the standardized mean difference based on *t*-test statistics or *F*-statistics with 1 degrees of freedom were employed (Lipsey and Wilson, 2001; Wilson, 2012). We ensured that our standardized mean difference score reflected the mean difference between immigrants under conditions of low versus high stereotype threat. Our standardized mean difference scores never represented an interaction effect (e.g., stereotype threat treatment by immigrant background). We did not consider such interaction effects because they can be driven – in part or completely – by stereotype lift effects (Walton and Cohen, 2003) among non-immigrant groups. We also did not consider multi-group comparisons; when the stereotype threat treatment involved more than one group (e.g., reflecting stereotype threat low, intermediate, and high) those two groups were compared that should theoretically exhibit maximum difference. In some studies several performance scores were reported (e.g., scores for two or more subtests of an intelligence test, Pellegrini, 2005; Wicherts et al., 2005). In order to preserve independence of effect sizes, we averaged the scores before they were included in the meta-analysis (Headrick, 2010).

### Coding of Study Characteristics

#### Sample Background

We distinguished two broader groups of studies based on their origin and sample: those that addressed stereotype threat among Latinos in the US (*k* = 11) and studies that focused on immigrants in Europe (*k* = 8). The latter studies were conducted in Austria, Belgium, France, Germany, or the Netherlands.

#### Circulation

Whereas nine studies were published in outlets of educational or social psychology, a majority of 10 studies was unpublished.

#### Age Group

All studies conducted in the US and four of the European studies investigated samples of young adults, typically consisting of undergraduates (*k* = 14). Five of the European studies investigated adolescents.

#### Stereotype Threat Treatment

Based on the distinction of stereotype threat-activating cues in the meta-analysis by Nguyen and Ryan (2008), out of the 19 experiments included in our meta-analysis, seven studies were found to have used indirect and subtle cues, five used moderately explicit cues, and seven used blatant stereotype threat-activating cues.

#### Dependent Measures

We distinguished studies that used measures of knowledge and abilities such as GRE-type tasks (verbal or maths, *k* = 13) from studies that used non-verbal measures of general cognitive functioning, such as (working) memory tests, or tests in the tradition of Raven’s progressive matrices (*k* = 6).

#### Group Categorization

There are different ways to distinguish immigrant group members from non-immigrants. Demographic surveys and statistical reports (e.g., OECD, 2010) typically rely on the place of birth of the individuals or their parents. Another common method is to ask individuals with which group they identify most. Studies were coded whether or not self-identification was the means to distinguish between immigrant group members or non-members (self-identification, *k* = 13).

### Comparison with Previous Meta-Analyses

Our dataset diverges remarkably from the data obtained in previous meta-analyses on stereotype threat. In addition to differences in the main aims and methodological approaches outlined above (focus on immigrants, quantification of stereotype threat main effects, and moderating variables), there are specific differences that appear noteworthy. We decided to meta-analyze the main effects or the simple main effects of the stereotype threat treatment on immigrants. As a consequence, the effect sizes integrated in our meta-analysis differ in part from the effect sizes reported in the meta-analysis by Nguyen and Ryan (2008). One previous meta-analysis (Nadler and Clark, 2011) identified six studies on stereotype threat effects among Latino samples in the US. We did not include two of these six studies in our analysis, because they did not meet our inclusion criteria. In one study (Stone, 2002) a negative stereotype with respect to White Americans was examined, Latinos served as a control group. In the second study (Good et al., 2003), treatment effects among Latino, Black, or White students were not distinguished. Thus, only four out of the 19 identified studies were already included in the meta-analysis by Nadler and Clark (2011).

## Results

### Meta-Analytic Results: Average Effects

#### Effect Size of the Stereotype Threat Treatment

The meta-analytic procedure followed the recommendations by Lipsey and Wilson (2001; Wilson, 2012). All standardized effect sizes were adjusted for small sample bias (Hedges, 1981). The inverse variance served as a weight that was allotted to each effect size (see Lipsey and Wilson, 2001, for the respective formulas). Negative effect sizes indicate a worse performance in the stereotype threat high than in the stereotype threat low condition (**Table 2**). All studies except for one (Wicherts et al., 2005, Study 1) had a negative effect size, indicating that in the great majority of studies the descriptive means followed the pattern expected from stereotype threat theory. The unweighted mean of the studies’ effect sizes amounted to *M* = -0.68 (SD = 0.54). With respect to outliers, the individual effect size of one study (Berjot et al., 2011) was around *M* ± 3 SD of the average unweighted mean effect size. No other study surpassed the threshold of *M* ± 2 SD. Results with and without this particular study were inspected.

**Table 2:** Study results and statistics.

Study no.	Immigrant sample size	SMD (d)	SE _SMD(d)_	w_iv_
1^a^	49	-0.41	0.29	11.96
2^b^	40	-1.45	0.36	7.92
3	44	-2.37	0.39	6.46
4^c^	50	-0.72	0.29	11.75
5	36	-0.47	0.34	8.75
6	126	-0.02	0.18	31.47
7	60	-0.70	0.27	14.13
8	47	-1.13	0.31	10.11
9^d^	34	-0.64	0.35	8.09
10	78	-0.21	0.23	19.26
11	60	-0.51	0.26	14.53
12^e,f^	20	-0.92	0.47	4.48
13^e^	56	-0.67	0.28	13.18
14	33	-0.54	0.35	7.96
15	44	-0.68	0.31	10.39
16	40	-0.64	0.32	9.51
17	80	-0.33	0.23	19.72
18^g^	25	-0.63	0.42	5.73
19	138	0.06	0.17	34.37


*SMD, standardized mean difference (after adjustment for small sample size bias, Hedges, 1981); w_iv,_ inverse variance weight.*

*^a^Effect size was derived from change score with additional information not included in the article: *r*_pre/post_ (control condition) = 0.85; *r*_pre/post_ (stereotype threat condition) = 0.66.*

*^b^Descriptives for men and women were averaged.*

*^c^Equal group size was presumed.*

*^d^“Control group” as low stereotype threat group.*

*^e^Only post-test scores were analyzed.*

*^f^ST+EM (effort meter) served as maximum ST-condition.*

*^g^Based on *t*-test results (p. 83) and reduced sample size.*

The weighted average effect of the stereotype threat manipulation over 19 independent effect sizes amounted to *d* = -0.63 for the random effects model (95% CI = -0.86; -0.40), SE = 0.12, *Z* = -5.36, *p* < 0.001, and *d* = -0.49 (95% CI = -0.61; -0.36) for the fixed effects model, SE = 0.06, *Z* = -7.68, *p* < 0.001. This indicates an average effect in support of stereotype threat theory among immigrant samples. This effect holds if the outlier study is excluded, random effects model; *d* = -0.52 (95% CI = -0.71; -0.34), SE = 0.09, *Z* = -5.58, *p* < 0.001, fixed effects model: *d* = -0.44 (95% CI = -0.56; -0.31), SE = 0.06, *Z* = -6.80, *p* < 0.001. According to the interpretation by Cohen (1988), the overall effect is medium in size. We further examined the homogeneity of our sample of effect sizes. With *Q*(18) = 57.04, *p* < 0.001, the effect sizes were significantly heterogeneous, highlighting the importance of subsequent moderator analyses.

#### Analysis of Sampling Bias and File-Drawer Analysis

Our goal in the meta-analysis was to include a maximum of studies, published or unpublished, written in English, Spanish, German, or French. More than half of our datasets originated from unpublished research. As reported in the previous section, these studies’ average effect is significant and moderate in size. Despite our efforts, however, it is unlikely that we were able to uncover every study conducted so far that would have met our criteria.

**FIGURE 1:**
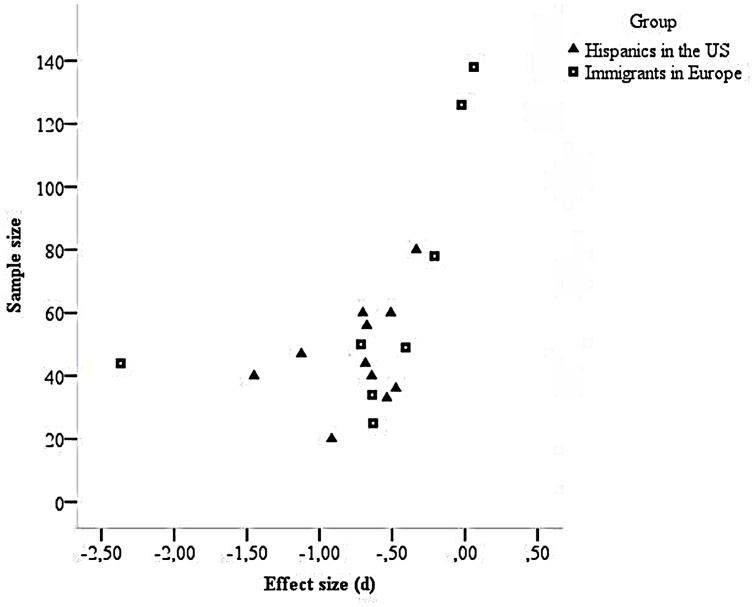
**Funnel plot based on effect size (d) and sample size.** In studies with negative effect sizes, low stereotype threat groups outperformed high stereotype threat groups.

In order to estimate a potential sampling bias in our set of studies we first plotted the meta-analytic data for a visual inspection (**Figure 1**). The funnel plot illustrates that the great majority of studies yielded a negative effect size, indicating that the direction of the effect was regularly in support of stereotype threat effects. It further shows that studies with larger samples were more likely to yield null effects. The plot points at a lack of small-sample studies with effects that do not support a stereotype threat hypothesis. One reason for this finding could be a selective reporting of small-scale studies. In order to gage the file drawer problem (Rosenthal, 1979), we first calculated the number of studies confirming the null hypothesis that would be needed to conclude that the effect is small. We used a formula by Orwin (1983; Lipsey and Wilson, 2001) and we set the limit of a small effect size at *d* = 0.2 (Cohen, 1988). This analysis yielded a fail-safe number of 46 unaccounted for studies in support of the null hypothesis that were needed to reduce the effect size to *d* = 0.2. When the limit was set at an arguably insubstantial average effect size of *d* = 0.1, a fail-safe number of 111 unaccounted for studies in support of the null hypothesis was identified.

Taken together, our sampling analysis pointed out a remarkable lack of null effects in small sample studies. If such studies were conducted, they were unavailable to us. A file-drawer analysis showed that the number of studies in support of the null hypothesis that were needed to change the average effect size to small or even to insubstantial is rather large. Thus, we conclude that the average effect size in support of a stereotype threat effect among people with an immigrant background is not severely challenged by potentially existing but unaccounted for studies. As a complement to our discussion on publication bias, the following moderator analyses included publication status (published versus unpublished) as one possibly influential factor.

### Meta-Analytic Results: Moderator Analyses

Due to the fact that the effect sizes were significantly heterogeneous, we inspected the influence of factors with theoretical relevance. This included the focused immigrant group (Latinos in the US versus immigrants in Europe), participants’ age, the experimental treatment, the dependent variable, and method of group categorization (see **Table 3**). Before turning to these conceptually relevant aspects, differences between published and unpublished studies are addressed.

**Table 3:** Summary of the moderator analyses, mixed effects model.

Variable	*k*	Between-groups analysis	Subgroup effect size	By group analysis
Circulation		*Q*(1) = 2.00, *p* = 0.157		
Unpublished	10		*d* = -0.47 (95% CI = -0.79; -0.15, SE = 0.16, *Z* = -2.91, *p* = 0.004)	*Q*(9) = 2.39, *p* = 0.984
Published	9		*d* = -0.81 (95% CI = -1.14; -0.47, SE = 0.17, *Z* = -4.67, *p* < 0.001)	*Q*(8) = 14.01, *p* = 0.082
Immigrant group		*Q*(1) = 0.79, *p* = 0.374		
Latina/o in the US	11		*d =* -0.71 (95% CI = -1.00; -0.42, E = 0.15, *Z* = -4.74, *p* < 0.001)	*Q*(10) = 4.06, *p* = 0.945
Immigrants in Europe	8		*d* = -0.51 (95% CI = -0.84; -0.18, SE = 0.17, *Z* = -3.01, *p* = 0.003)	*Q*(7) = 15.37, *p* = 0.032
Age group		*Q*(1) = 9.97, *p* = 0.002		
Adolescents	5		*d* = -0.17 (95% CI = -0.48; 0.15, SE = 0.16, *Z* = -1.05, *p* = 0.294)	*Q*(4) = 1.99, *p* = 0.738
Adults	14		*d* = -0.79 (95% CI = -1.01; -0.57, SE = 0.11, *Z* = -7.12, *p* < 0.001)	*Q*(13) = 17.57, *p* = 0.175
Stereotype threat treatment		*Q*(2) = 5.98, *p* = 0.050		
Subtle	7		*d* = -0.62 (95% CI = -0.96; -0.27, SE = 0.18, *Z* = -3.51, *p* < 0.001)	*Q*(6) = 3.81, *p* = 0.702
Moderate	5		*d* = -1.05 (95% CI = -1.49; -0.62, SE = 0.22, *Z* = -4.70, *p* < 0.001)	*Q*(4) = 9.95, *p* = 0.041
Blatant	7		*d* = -0.36 (95% CI = -0.70; -0.04, SE = 0.17, *Z* = -2.19, *p* = 0.029)	*Q*(6) = 2.41, *p* = 0.879
Dependent measures		*Q*(1) = 2.94, *p* = 0.086		
Non-verbal, fluid intelligence tasks or memory	6		*d* = -0.93 (95% CI = -1.37; -0.48, SE = 0.23, *Z* = -4.05, *p* < 0.001)	*Q*(5) = 9.75, *p* = 0.083
GRE-like (verbal, maths)	13		*d* = -0.50 (95% CI = -0.75; -0.25, SE = 0.13, *Z* = -3.92, *p* < 0.001)	*Q*(12) = 8.59, *p* = 0.737
Group categorization		*Q*(1) = 5.63, *p* = 0.018		
Immigrant status not self-identified only	6		*d* = -0.28 (95% CI = -0.61; 0.05, SE = 0.17, *Z* = -1.69, *p* = 0.090)	*Q*(5) = 3.48, *p* = 0.626
Immigrant status self-identified	13		*d* = -0.78 (95% CI = -1.02; -0.54, SE = 0.12, *Z* = -6.29, *p* < 0.001)	*Q*12) = 8.59, *p* = 0.192

#### Publication

More than half of our studies were unpublished, so it seemed warranted to contrast published with non-published effects. The moderator analysis yielded a non-significant difference, *Q*(1) = 2.00, *p* = 0.157, indicating that the effect sizes between both groups did not significantly differ. This result holds with or without the study by Berjot et al. (2011). The average standardized mean differences were significant both for published studies, *d* = -0.81, SE = 0.17, *p* < 0.001, as well as for unpublished research *d* = -0.47, SE = 0.16, *p* = 0.004.

#### Latinos in the US versus Immigrants in Europe

One main goal of the meta-analysis was to examine potential differences in effect size between studies conducted with Latino samples in the US and immigrants in Europe in order to gage whether stereotype threat is a sufficiently replicated phenomenon with immigrant samples outside the US.

Based on the 19 studies, there were no significant differences between the two groups, *Q*(1) = 0.79, *p* = 0.374. Separate calculations showed that the average effects were significant for Latinos in the US, *d* = -0.71, SE = 0.15, *p* < 0.001, as well as for immigrants in Europe *d* = -0.50, SE = 0.17, *p* = 0.003, indicating equivalent support for stereotype threat theory. Studies with Latinos in the US showed homogeneous effect sizes, *Q*(10) = 4.06, *p* = 0.945. For studies with immigrants in Europe, a significant score, *Q*(7) = 15.37, *p* = 0.032, pointed at remarkable heterogeneity. In a subsequent step, we tried to examine whether the heterogeneity could be attributed to the one study with an exceptionally large effect size (Berjot et al., 2011) which was situated in Europe. Would the significance of average mean difference scores for European studies still hold, if this study was eliminated from the dataset? The re-analysis yielded a significant moderation effect of immigrant group, *Q*(1) = 9.12, *p* = 0.003, indicating that the effect sizes for studies conducted in Europe were lower than the effect sizes for US studies. When the extreme score of the study by Berjot et al. (2011) was excluded, the average standardized mean difference dropped for the European study sample, but was still significant, *d* = -0.24, SE = 0.11, *p* = 0.026. This additional analysis pointed to consistent stereotype threat effects for both US Latino and European immigrant samples, even if the effects obtained from the latter group might be weaker than those obtained from Latinos in the US.

All results for the following moderator analyses hold with or without the study by Berjot et al. (2011), so they are based on the full sample.

#### Age Group

As noted in the method section, the five datasets on adolescent samples under threat came from European studies. The moderator analysis revealed a larger effect among adults (which were mostly undergraduates) than among adolescents, *Q*(1) = 9.97, *p* = 0.002. The present data of five independent studies allow no clear conclusion on whether stereotype threat affects immigrant adolescents or not, *d* = -0.17, *p* = 0.294.

#### Treatment Type

In line with the findings from a previous meta-analysis (Nguyen and Ryan, 2008), moderately blatant stereotype threat treatments yielded the largest results [the difference between the three treatment-groups was trend-significant, *Q*(2) = 5.98, *p* = 0.050], but all types of treatment were effective (see **Table 3**).

#### Dependent Measures

We identified a trend-significant difference between the dependent measures, *Q*(1) = 2.94, *p* = 0.086, suggesting that studies which used non-verbal, fluid intelligence measures (memory tasks, RPM-like tasks) obtained somewhat stronger effects than studies that used GRE-like tasks. The average influence of the stereotype threat treatment was significant for both groups of dependent variables.

#### Group Categorization

In order to specify immigrant status, thirteen studies relied on self-identification while six studies used other procedures, including, for example, the parents’ birthplace. In the latter studies, some participants might be categorized into the immigrant group whereas they would have self-identified to be a majority group member, resulting in the violation of a relevant precondition for stereotype threat to occur, namely the identification with the stereotyped target group. Studies in which immigrant group members self-identified to belong to this group yielded larger effect sizes than studies in which self-identification was no prerequisite, *Q*(1) = 5.63, *p* = 0.018. This is in line with the assumption that a stereotype threat treatment might fail for individuals who technically fall into the immigrant group category, but self-identify to be a majority group member.

### Stereotype Threat and Identity Strength

Some research conducted outside the stereotype threat framework identified the strength of an immigrant’s psychological ties to his or her ethnic group to be an adaptive factor that attenuates the influence of ethnicity-related stressors (e.g., Umaña-Taylor and Updegraff, 2007; Armenta and Hunt, 2009). Within the stereotype threat framework, however, identity strength is considered to increase threat and to reduce cognitive performance (Schmader et al., 2008).

Unfortunately, few studies in our sample addressed identity strength as a potential moderating factor. In the experiment by Armenta (2010), identity strength was measured prior to the main experimental session. The participants worked on the affirmation and belonging subscale of the Multi-Group Ethnic Identity Measure (MEIM, Phinney, 1992). This subscale measures an immigrant’s attachment to his or her ethnic group. The results yielded a significant three-way interaction between group (Asian versus Latino), the activation of ethnicity (supposedly eliciting threat among Latinos) and ethnic identity strength. For Latinos, a stereotype threat treatment effect emerged among those with high scores on ethnic identity strength, while the treatment had no influence on the performance of those with low scores.

In a similar vein, one experiment by Schultz et al. (unpublished manuscript, Study 2) revealed a significant interaction between stereotype threat condition and ethnic identity strength. Latino participants with a strong ethnic identity scored lower on the verbal exam in the threat condition. In contrast, higher ethnic identity scores predicted higher verbal exam scores in the control condition. In a follow-up study by Schultz et al. (unpublished manuscript, Study 3) only a main effect for ethnic identity strength was identified, suggesting that this measure was negatively related to performance across groups. A moderation effect between stereotype threat treatment and identity strength could not be established, the stereotype threat effects were independent of ethnic identity strength.

In the fourth relevant experiment, immigrants from various origins in Austria (most frequent were Kosovo, Bosnia, and Turkey) were exposed to anti-immigrant political ads or neutral political ads (Appel, 2012). The MEIM was used to measure ethnic identity strength after the treatment and the dependent variables. No significant interaction between ethnic identity strength and the treatment was found. However, a simple slope analysis indicated that in this experiment, ethnic identity might have worked as a buffer. For participants low in ethnic identity strength a significant influence of the treatment was observed, whereas participants with high scores on ethnic identity strength were unaffected by the treatment.

In sum, evidence for the role of ethnic identity strength in stereotype threat studies with immigrant samples is sparse and controversial; two experiments observed a significant moderation effect whereas two other studies are inconclusive, one even suggesting that ethnic identity strength can serve as a buffer. It further needs to be noted that, thus far, no study has examined the role of residence culture identity strength for immigrants in situations of stereotype threat.

## Discussion

Historically, many immigrant groups have been faced with negative achievement related stereotypes. In the 1750s, for example, Benjamin Franklin was known for his skepticism regarding German immigrants in Pennsylvania, the “swarthy” “Palatine Boors” (Franklin in Labaree, 1959) who at that time were widely perceived to be lazy, illiterate, and reluctant to assimilate (Feer, 1952). Today, several immigrant groups in most parts of the world underperform in educational settings. Stereotype threat theory posits that negative stereotypes about one’s group can elicit an extra pressure not to fail which leads to cognitive underperformance. Thus, stereotype threat could explain a substantial part of the immigrant achievement gap, one of the arguably most pressing problems for educational research and practice. This meta-analytic review summarized experiments in which immigrants worked on a test of cognitive performance under conditions of low versus high stereotype threat.

Our meta-analysis of 19 independent effect sizes show that the average stereotype threat treatment effect is substantial and significant. This result holds for stereotype threat effects among US samples of Latino background (*k* = 11) as well as among immigrant samples in Europe with various ethnic backgrounds (*k* = 8). All studies conducted in the US were based on young adults (mostly undergraduates). The effects found for adults were larger than for samples of immigrant children and adolescents (*k* = 5, all from Europe). Our findings showed that all treatment types (subtle, moderately explicit, blatant) were effectively altering immigrants’ performance. Likewise, studies that used performance measures with a rather strong learnt knowledge component (GRE-type) and studies that used performance measures with a rather strong fluid intelligence component (memory tasks, RPM-typed tasks) yielded significant stereotype threat effects. Over half of our datasets (*k* = 10) originated from unpublished or yet-to-be-published research. For both types of studies, published or unpublished, substantial and significant average effect sizes were found. A fail-safe analysis (Orwin, 1983; Lipsey and Wilson, 2001) indicated that for the effect size to drop to *d* = 0.2, an additional 46 unaccounted for studies were needed, and for the effect size to drop to *d* = 0.1, the number of unaccounted for studies amounted to 111.

The comparison between published versus unpublished research and the fail-safe analysis suggest that the sample of studies is not strongly influenced by publication or reporting bias. The funnel plot, however, shows a remarkable pattern of asymmetry: studies with smaller samples tend to yield larger effect sizes in support of stereotype threat than studies with larger samples. There are at least two possible explanations for such an asymmetry. First of all, the corpus of retrievable studies could be subject to a systematic bias in publication and – regarding unpublished research – in accessibility. Publication bias is common in the social sciences, as statistically significant results are more likely to be published than non-significant results, particularly if a small sample study is underpowered. Regarding unpublished research, studies that are underpowered and yield no significant differences are more likely to leave almost no trace that they were ever conducted. Such studies might not be included in a dissertation, they may not be presented at conferences, and so on.

Second, the asymmetrical pattern could be due to heterogeneity among the studies. Studies with larger samples could have a different design or method than studies with smaller samples. For example, small sample studies might be based on individual or small group experimental sessions whereas large sample studies might be based on large-group experimental sessions. The latter procedure, in turn, could increase error variance and therefore decrease the likelihood of identifying significant treatment effects. Due to the fact that few study reports included the number of participants for each experimental session, a systematic analysis of this explanation is impossible. However, our moderator analysis identified the method with which immigrant status was assessed to be an influential factor regarding the studies’ results. The three studies with the largest sample sizes were also among the four studies with the least support for stereotype threat effects. All three studies were among European studies that determined immigrant status with the help of demographic categories, rather than self-identification. For example, Wicherts et al. (2005) used the participants’ parents’ place of birth to decide whether a participant was considered as a member of the residence culture group (Dutch) or the immigrant group. If at least one parent was born outside the Netherlands, the participant would fall into the immigrant category. The authors further report that 17% of those categorized as immigrants self-identified strongly to be Dutch (rather than identifying with Antilles/Suriname, Turkey, or Morocco). Another 16% identified both with the Netherlands and their immigrant group. As a consequence, the stereotype threat manipulation might have failed to elicit stereotype threat among those who self-identified to be Dutch which amounts to one third of the study sample. In summary, the fact that studies with larger samples yielded less support for stereotype threat effects is likely due to a combination of publication/accessibility bias and heterogeneity of the studies regarding immigrant status assessment.

Our analyses as a whole, including the fail-safe-n analysis and the substantial effect of unpublished research lead to the conclusion that the detrimental effect of stereotype threat on immigrants’ performance has been convincingly established by prior research. Therefore, reducing the detrimental impact of stereotype threat for negatively stereotyped immigrants in educational settings appears to be an important objective for research and educators alike.

### Limitations and Future Research Directions

As with all studies, the present review and meta-analysis has limitations that need to be noted. First, our aim in the current meta-analysis was to identify main effects of the stereotype threat treatment. According to stereotype threat theory and research (Schmader et al., 2008), these main effects can be subject to moderation effects identified with the help of additional experimental factors or questionnaires. Due to its key importance in acculturation research we had a closer look on ethnic identity strength as a variable that might affect the magnitude (and direction) of stereotype threat effects. Other moderators were idiosyncratic to single studies. This review and meta-analysis will likely inspire future research rather than dispirit researchers, because exciting questions are still to be answered (cf. Chan and Arvey, 2012). Future research is encouraged to further clarify variations in stereotype threat effects. To name an example, the length of time spent in a country (or generational differences) should be examined more closely. So far, studies within the stereotype threat framework have primarily addressed social groups that have a life-time of exposure to being stereotyped (because of their gender or the color of their skin). The finding that stereotype threat also is found among immigrants raises the question of how much exposure to being stereotyped is necessary for the detrimental effects of stereotypes to become visible, which is a highly important question from a theoretical point of view. Heterogeneity among immigrant groups might be of importance here. While some may be able to merge into the new culture and become less affected by stereotypes over time, for others – due to their merging in a stereotyped subgroup – the susceptibility to stereotype threat may increase (Deaux et al., 2007). The visibility of belonging to an immigrant group might be but one factor that plays a role.

Second, our focus was on performance measures. Although stereotype threat is best known for its influence in test-taking situations, theory and recent research indicate that stereotype threat might affect individuals prior to test-taking (Appel and Kronberger, 2012); that it can lead to short-term (Steele, 1992, 1997; Davies et al., 2002) and long-term disidentification (Osborne and Walker, 2006; Kronberger and Horwath, 2013), and less successful preparation and learning (Rydell et al., 2010; Appel et al., 2011). Stereotype threat studies on domain identification, career choice or learning are generally rare, and we identified no study on any of these topics that involved immigrants.

Third, the contribution of this meta-analysis regarding the immigrant achievement gap is indirect. The present meta-analysis did not compare the performance of immigrants versus non-immigrants. Still, our results suggest that immigrants’ performance varies depending on whether stereotype threat is increased or reduced, which in turn can increase or decrease the distance to non-immigrants’ performance.

Fourth, the specific content of stereotypes directed at different groups of immigrants demands for closer inspection, particularly in non-US contexts; the *Stereotype Content Model* (Lee and Fiske, 2006) identifies subgroups and their perception in society along the two dimensions of warmth and competence. This approach seems promising for future attempts to clarify the relationship between stereotype content, status of an immigrant group in society, and the effects of stereotype threat.

Finally, despite some efforts to include as many studies as possible from around the world, our search strategy (focus on English reports, etc.) may have privileged studies on immigration to the US and Europe. There clearly is a need to address the topic in other parts of the world as well.

Investigating stereotype threat among immigrants touches the huge field of research on immigrants’ identity and acculturation processes. Regarding questions of identity, there are several points to be made that may inform future research. First, the present research indicates that researchers need to thoroughly reflect about their method to assess immigrant status. Self-identification and (parent’s) birthplace methods may at times yield conflicting results. Second, ethnic identity strength has been conceived as a one- or two-dimensional concept, including the sub-factors commitment and exploration (e.g., Phinney, 1992). In future research it could be useful to consider additional dimensions and facets, for example, private regard of one’s ethnic culture or remigration prospects. So far, this aspect has not been sufficiently addressed in stereotype threat research. Third, there is a research history of describing immigrant identity along the lines of both ethnic identity and residence culture identity (sometimes addressed as ‘national identity’). Future research on stereotype threat among immigrants is advised to examine both identity dimensions. Possibly, individuals with a strong residence culture identity are less affected by potentially threatening cues, independent of their ethnic identity strength. Immigrants with a strong residence culture identity might at times switch between identities, as they can be regarded *bicultural*, being able to draw from resources of two (cultural) backgrounds. There is evidence that it is possible to activate a particular identity in people with a bicultural identity (cf. Hong et al., 2000). Consequently, people then think, feel, and act according to the activated identity. If this is the case, active cultural frame switching could protect bicultural immigrants in situations of stereotype threat (Shih et al., 1999). As a consequence, stereotype threat vulnerability might be decreased due to a strong identification with the residence culture.

### Implications

Our review and meta-analysis suggests that stereotype threat is a psychological predicament with detrimental consequences on immigrants’ performance. Thus, reducing the negative influence of stereotype threat could help closing the achievement gap. Stronger cognitive performance and educational success can increase the opportunities of immigrants to participate in their resident societies. Moreover, closing the immigrant achievement gap could be one important key to the future prosperity of societies. According to the (World Economic Forum [WEF], 2011) many countries in the Northern hemisphere will soon be faced with shortages of proficient workforce due to an aging population and insufficient education. According to this economic data, Western Europe, for example, is required to add over 45 million people to its talent base by 2030 in order to sustain economic growth. As well-educated personnel is becoming increasingly scarce, companies and countries “will need to be creative in identifying under-utilized or underdeveloped pools of talent” (p. 19).

Previous research on gender and race identified strategies to reduce the negative influence of stereotype threat (e.g., for an overview see Gehlbach, 2010; Yeager and Walton, 2011; Cohen et al., 2012). A main approach is reducing the salience of the negative link between belonging to a minority group and learning and performance at school. As our meta-analytic findings demonstrate, even subtle cues, like asking negatively stereotyped immigrant students about their ethnic background prior to a task, can decrease performance to the same extent as blatant cues, such as stating the cognitive inferiority of a group. This aspect is crucial for practical implications in school or work environments, where immigrants might constantly experience the fear to underperform due to subtle stereotype activating cues, even if blatant cues (e.g., use of discriminatory or sexist language) are largely frowned upon and regarded as politically incorrect. The resulting actual underperformance might then reinforce the negative stereotype (self-fulfilling prophecy), which makes it hard to break the cycle. Moreover, valuing diversity and increasing the visibility of minority group members who may be fellow students or teachers decreases stereotype threat effects (e.g., Purdie-Vaughns et al., 2008; Carrell et al., 2010). Stereotyped students can further profit from successful role models (e.g., McIntyre et al., 2003), but perceived dissimilarities between oneself and the role model might reduce the influence or even yield detrimental effects (e.g., Cheryan et al., 2011; Asgari et al., 2012). An intriguing line of research showed that brief classroom interventions that highlight students’ core personal values can improve the performance of negatively stereotyped students (e.g., Cohen et al., 2006, 2009; Miyake et al., 2010; cf., Yeager and Walton, 2011).

The attractiveness of the social-psychological strategies to increase school performance lies in the fact that small and easy to implement interventions can result in considerable improvements for those concerned. To date, very little research on these strategies has focused on immigrant students. However, our review on stereotype threat suggests that these strategies, applied to the group of immigrants, could reduce the detrimental effects of stereotype threat and could contribute to a better achievement of immigrant students. In line with this assumption, a field experiment on values affirmation provides initial evidence on the effectiveness of these strategies for immigrant students (Sherman et al., 2013). In addition to academic success, research on stereotype threat spillover (Inzlicht and Kang, 2010; Aronson et al., 2013) suggests that interventions to combat stereotype threat might effectively reduce immigrant-majority member gaps in fields such as health or aggression. In summary our analyses suggest that immigrant students in many parts of the world are confronted with a stereotype threat that endangers them to perform below their potential. However, our analyses also suggest that there is considerable need for further research to better understand how this threat actually works and how it can be mitigated.

## Conflict of Interest Statement

The authors declare that the research was conducted in the absence of any commercial or financial relationships that could be construed as a potential conflict of interest.

## Footnotes

1 Note that a longer duration of time spent in a country does not necessarily need to make the situation better for those stigmatized immigrants. Second generation Afro-Caribbeans, for example, were found to be *more* rather than less susceptible to stereotype threat compared to first generation immigrants, because stereotypes toward African Americans applied (Deaux et al., 2007; see also Waters, 1999).

2 Stereotype threat further depends on the extent individuals are generally aware of the negative stereotype (e.g., stigma consciousness, Brown and Pinel, 2003) and on the situational cues that activate or deactivate negative stereotypes (e.g., Steele and Aronson, 1995; Spencer et al., 1999).

3 We further made sure that Arab^∗^ and African^∗^ immigrants to Europe were covered by the search procedure; only the group of African Americans was to be excluded. No additional studies were found.
